# S72. PRO-SOCIAL PROSPECTIVE MEMORY PERFORMANCE IS ASSOCIATED WITH PLASMA OXYTOCIN LEVEL IN SEXUALLY DIMORPHIC WAY IN FIRST-DEGREE RELATIVES OF SCHIZOPHRENIA

**DOI:** 10.1093/schbul/sby018.859

**Published:** 2018-04-01

**Authors:** Fuchun Zhou, Lan Xiong, Ping Zhou, Yu-Tao Xiang, Chuan-Yue Wang

**Affiliations:** 1 Beijing Anding Hospital, Capital Medical University; 2 Université de Montréal; 3 Shenzhen Kangning Hospital; 4 University of Macau

## Abstract

**Background:**

Prospective memory (PM) in real world is mostly societal and it often involves activities in which the individual has to remember to do something for others, which is also called pro-social PM. The neuropeptide oxytocin has been implicated in social cognition and social interaction in a number of studies. The aim of the present study was to investigate the correlation between pro-social PM performance and plasma oxytocin level in first-episode schizophrenic patients (FES), first-degree relatives (FDRs) of schizophrenia, and healthy controls (HCs). In addition, we also tried to explore the sexually dimorphic feature in the interactions of above-mentioned factors.

**Methods:**

Forty-six FES patients, 41 non-psychotic FDRs of patients with chronic schizophrenia (unrelated to the FES group) and 54 HCs were studied. Pro-social time-based prospective memory (TBPM) and event-based prospective memory (EBPM) performance were assessed with the Chinese version of the Cambridge Prospective Memory Test (C-CAMPROMPT). A serial of tests reflecting retrospective memory and executive functions were also administrated. Plasma oxytocin levels were determined by radioimmunoassay using a RIA kit.

**Results:**

(1) There were significant differences in performance between FES, FDRs, and HCs with respect both TBPM and EBPM, even after controlling for age, sex and education level by analysis of covariance (ANCOVA). We found significant group*sex interaction only regarding TBPM (F(2, 133)=4.8, p=0.01). Female HCs performed significantly poorer than male HCs on TBPM (11.1 ± 5.5 vs 14.4 ± 4.8, t=-2.3, p=0.026). However, this sexually dimorphic trend was not seen in either FES (9.0 ± 5.1 vs 7.5 ± 5.1, t=1.0, p=0.34) or FDRs (12.3 ± 3.5 vs 11.1 ± 4.4, t=1.0, p=0.3). (2) A significant group*sex interaction was also revealed with regard to plasma oxytocin level (F(2, 134)=4.1, p=0.018). In HCs, females exhibited significantly higher plasma oxytocin level than males (62.2 ± 28.3 pg/ml vs 44.4 ± 20.9 pg/ml, t=2.5, p=0.015). But this sexually dimorphic feature did not appear in FES (58.3 ± 20.1pg/ml vs 59.6 ± 19.4 pg/ml, t=-0.2, p=0.822) or FDRs (60.4 ± 19.2 pg/ml vs 74.0 ± 46.0 pg/ml, t=-1.2, p=0.230). In addition, there was a significant difference in plasma oxytocin level between the three groups only in male. Post-hoc analyses suggest male FDRs exhibited significant higher plasma oxytocin level than male HCs. (3) After controlling age and education level, partial correlation analysis indicated higher plasma oxytocin levels to be significantly associated with higher TBPM scores in FDRs (r=0.39, p=0.015). In order to determine the origin of this correlation, we further conducted 2 separate partial correlation analyses in males and females, still with age and education level as controlled variables. The correlation between plasma oxytocin level and TBPM remained significant in male FDRs (r=0.5, p=0.021) but disappeared in female FDRs (r=0.2, p=0.434).

**Discussion:**

In the present study, we found the pre-existing sex-specific patterns (as in HCs) of plasma oxytocin level and TBPM were substantially disrupted in FES and FDRs. Moreover, a significant association between plasma oxytocin levels and PM was only found in FDRs, and only male FDRs contribute to this significant association, suggesting oxytocin may play an important role regulating pro-social PM in FDRs in sexually dimorphic way.

Longitudinal studies with larger sample size and measurement of oxytocin receptor function and genetic variations should be conducted in the future.

